# High Thyroid Stimulating Hormone Level Is Associated With Hyperandrogenism in Euthyroid Polycystic Ovary Syndrome (PCOS) Women, Independent of Age, BMI, and Thyroid Autoimmunity: A Cross-Sectional Analysis

**DOI:** 10.3389/fendo.2019.00222

**Published:** 2019-04-10

**Authors:** Jie Cai, Yi Zhang, Yuying Wang, Shengxian Li, Lihua Wang, Jun Zheng, Yihong Jiang, Ying Dong, Huan Zhou, Yaomin Hu, Jing Ma, Wei Liu, Tao Tao

**Affiliations:** ^1^Division of Endocrinology and Metabolism, Department of Internal Medicine, Renji Hospital, School of Medicine, Shanghai Jiaotong University, Pudong, China; ^2^Shanghai Key Laboratory for Assisted Reproduction and Reproductive Genetics, Center for Reproductive Medicine, Renji Hospital, School of Medicine, Shanghai Jiaotong University, Pudong, China

**Keywords:** thyroid stimulating hormone, polycystic ovary syndrome, dyslipidemia, hyperandrogenism, euthyroid

## Abstract

**Background:** Infertility and dyslipidemia are frequently present in both women with polycystic ovary syndrome (PCOS) and subjects with thyroid dysfunction. Limited study regarding the association between thyroid stimulating hormone (TSH) level and phenotypes in euthyroid PCOS women. We aimed to determine whether the variation of TSH level associates with phenotypes in euthyroid PCOS patients.

**Methods:** Cross-sectional study including 600 PCOS and 200 age, body mass index (BMI), and thyroid autoimmunity-matched Chinese women from Renji hospital, Shanghai Jiaotong university during January 2010 and August 2018. The anthropometric and serum biochemical parameters related to TSH, thyroid autoimmunity, lipid profiles, and sex steroids were detected.

**Results:** The TSH level is higher in (2.29 ± 1.24 vs. 1.86 ± 0.90 mu/L, *p* < 0.001) in PCOS than controls. In euthyroid PCOS patients, TSH, TG, TC, LDL-c, and apoB level increased from non-hyperandrogenism (nonHA) to HA group (all *p* < 0.05). TSH level is positively associated with TG, apoB, free T, FAI, and negatively associated with apoA (all *p* < 0.05). The percentage of HA increased from TSH level (57.93% in TSH < = 2.5 group vs. 69.46% in TSH > 2.5 mU/L group, *p* = 0.006). HA phenotype is increased with TSH level independently of age, BMI, WC, LDL-C. Besides, in multivariate logistic regression analysis TSH and TG significantly associated with HA phenotype.

**Conclusions:** Higher TSH level is associated with increased prevalence of HA phenotype independent of age, BMI and thyroid autoimmunity in euthyroid PCOS.

## Introduction

Polycystic ovary syndrome (PCOS) is the most common endocrine disorder with systemic metabolic manifestations and neuroendocrine-immunity disturbance in women of reproductive age (1). It is characterized by hyperandrogenism (HA)/hirsutism, oligo- or amenorrhea, and polycystic ovaries (PCO). PCOS is also a heterogeneous disorder that affects many body functions, resulting in several health complications, including infertility, menstrual dysfunction, hirsutism, acne, obesity, metabolic syndrome as well as autoimmune disease.

Thyroid hormone disorders or thyroid autoimmunity is associated with increased risk of infertile, spontaneous miscarriage, preterm delivery, and metabolic dysfunctions, which is also commonly observed in PCOS (2, 3). In PCOS population, the prevalence of subclinical hypothyroidism (SCH) and thyroid autoimmunity are reported higher than that for women in general (4–6). Emerging studies have investigated the association between thyroid function/thyroid autoimmunity and metabolic parameters in PCOS, especially in dyslipidemia and insulin resistance. SCH is observed in PCOS women and has been found to be associated with hyperlipidemia and affect pregnancy rate in both PCOS and general population (7–11). Bakker et al. reported serum thyroid stimulating hormone (TSH) value is associated with a higher risk for dyslipidemia and severe cardiovascular risk factors (12). Our previous study analyzed TSH level and lipid profile in PCOS population, suggesting the cut-off point of TSH is 4.07 mU/L for elevated LDL-c risk (11). Thyroid hormones may also act as insulin agonists in muscle and as antagonists in the liver, so deficiency of thyroid hormones may lead to a decrease in glucose production and utilization (13–15). So some authors have considered insulin resistance (IR), which has been considered to be the principal factor in the pathogenesis of PCOS, as a consequence of hypothyroidism (1, 16).

Thyroid hormones not only plays an important role in regulating metabolism but also in reproductive health. Both thyroid receptor and TSH receptor are expressed in ovary, uterus and widely expressed in the feto-maternal unit during implantation (1). Deficiency of thyroid hormones may affect gonadal function and fertility, leading to delayed puberty onset and anovulatory cycles (17). SCH is associated with body weight gain, sex hormone-binding globulin (SHBG) increase, androstenedione to testosterone conversion increase, and aromatization to estradiol (18). TSH has been described as the most sensitive parameters for detecting minor degrees of primary thyroid hormone deficiency (18). But there is a controversy on treatment threshold based on TSH value in infertile women. According to 2017 American thyroid association (ATA) guideline, evidences suggesting TSH 4.0 mU/L instead of 2.5 mU/L as treatment threshold of levothyroxine (L-T4) in women before or in pregnancy (18). While in those undergoing *in-vitro* fertilization (IVF) or intracytoplasmic sperm injection (ICSI) ATA guideline suggest they treated with L-T4 and goal of treatment is to achieve TSH concentration <2.5 mU/L. That indicating in infertile women, there might has underlying subtle hypothyroidism even in those with normal thyroid function. Although PCOS is the most common cause in women with infertility, rare studies investigate the association of TSH and HA in euthyroid PCOS population.

In this present study, we hypothesized that underlying subtle hypothyroidism in PCOS would be associated with HA in women with PCOS. The aim of the present study was to investigate the relationship of TSH and HA phenotypes between PCOS and age, body mass index (BMI), and thyroid autoimmunity matched euthyroid controls.

## Materials and Methods

### Subjects

We consecutively recruited participants who attended outpatient endocrine clinics of Renji hospital for investigation of oligo- or amenorrhea, fertility problems, hirsutism, or acne during January 2010 and August 2018. All the subjects were non-smokers from eastern China. Age, BMI and thyroid-autoimmunity matched control and PCOS subjects were included by a ratio of 1:3. In brief, PCOS and control women were stratified by age, BMI and the presence of autoimmune thyroiditis (AIT). Totally 600 PCOS (age: 27.67 ± 5.21 years, range: 14–50 years, BMI: 26.44 ± 5.69 kg/m^2^, range: 14.89–50.59 kg/m^2^) and 200 healthy control women (age: 28.26 ± 5.29 years, range: 14–49 years; BMI: 26.72 ± 5.37 kg/m^2^, range: 15.02–50.09 kg/m^2^) are analyzed. All subjects were received thyroid autoimmune antibody measurement and thyroid USG evaluation. Those with TSH level out of reference range (0.25–5.0 mIU/L), took thyroid treatment in last 6 weeks, or had thyroid, pituitary surgery history were excluded. The presence of either thyroid peroxidase (TPO) or thyroglobulin antibodies is defined as AIT. Euthyroidism is defined as TSH level within the reference range. The PCOS diagnosis was based on the revised Rotterdam 2003 criteria. These include the following: (i) clinical and/or biochemical signs of hyperandrogenemia; (ii) oligomenorrhoea or anovulation; and (iii) findings of polycystic ovaries by ultrasound (19). All patients of other related disorders should be excluded prior to examination, including non-classical 21-hydroxylase-deficient adrenal hyperplasia, hyperprolactinemia, thyroid dysfunction, Cushing's syndrome, or androgen-producing tumors (20). A free androgen index (FAI) >7 was considered diagnostic of hyperandrogenemia (HA) (21). PCOS was divided into four phenotypes: oligo/amenorrhea + HA (O + HA), polycystic ovaries + HA (PCO + HA), O + PCO, and O + PCO + HA. Subjects in the control group were nonHA healthy women and serum total testosterone values ≤0.6 ng/ml. Moreover, all of them had normal ovulation cycles and ovarian appearance in ultrasound examinations. All the study evaluations and procedures were conducted in accordance with the guidelines of the Helsinki Declaration on human experimentation. Written informed consent was obtained from all subjects and this study is approved by the Ethical Committees of Renji Hospital.

### Anthropometric Measurements

We used a digital scale and a stadiometer to measure the height and weight of each subject to the nearest 0.1 cm and 0.1 kg, respectively. The BMI was calculated as the body weight (kg) divided by the height (m) squared. The waist circumference (WC) was measured to the nearest 0.1 cm by placing measuring tape around the body in a horizontal position at a level midway between the lower rib margin and the iliac crest.

### Laboratory Assays

All the laboratory evaluations were performed on subjects in the fasting state between 7:00 a.m. and 8:00 a.m. during day 2 to 5 of the spontaneous menstrual cycle. If the patient had amenorrhea for more than 3 months, the examination was performed during a bleeding episode after progestin withdrawal. The blood samples were stored at 4°C on the day of the collection. All measurements, including total cholesterol (TC), triglyceride (TG), high-density lipoprotein cholesterol (HDL-c), and low-density lipoprotein cholesterol (LDL-c) were performed with Roche reagents (D 2400 and E 170 Modular Analytics modules with Roche/Hitachi analyzers; Roche Diagnostics).Analysis of thyrotropin (TSH), luteinizing hormone (LH), follicle stimulating hormone (FSH), total testosterone (T), progesterone (P), estradiol (E2), prolactin (PRL), sex hormone binding globulin (SHBG), and sulfated dehydroepiandrosterone (DHEA-S) were detected by chemiluminescence (Elecsys Auto analyzer, Roche Diagnostics). The FAI level was calculated as [(total testosterone × 100)/SHBG] (21).

### Statistical Analysis

All statistical analyses were performed using SPSS software version 17.0 (SPSS, Chicago, IL, USA). The PCOS patients were further divided into two groups on the basis of TSH level: Group 1: 0.25 ≤ TSH ≤ 2.5 mIU/L, Group 2: 2.5 < TSH ≤ 5.0 mIU/L. The data were presented as the mean ± SD, except for skewed variables, which were presented as the median with the interquartile range (IQR) given in parentheses. Ln transformation were conducted to achieve normal distribution for skewed variables. Student's *t*-test or Mann–Whitney *U*-test was used for the comparisons of continuous data among groups, whereas the Chi-squared test was used for the comparisons of categorical variables. Spearman correlation analysis were used to detect the associations between TSH level, lipid profiles and the present of HA. Multiple logistic regression analysis was used to assess the odds ratio (OR) for the presence of HA in PCOS subjects. Factors that were significantly associated with HA in the univariate analyses were entered in the multivariate regression model. A two-tailed test was applied, and a *P*-value of <0.05 was considered statistically significant.

## Results

### Clinical and Biochemical Characteristics of PCOS and Controls

General demographic and laboratory characteristics of the study cohort are summarized in Table 1. The mean age of our population was 27.67 ± 5.21 years in PCOS women and 28.26 ± 5.29 years in control. Among all participants, 153 were autoimmune-thyroiditis (AIT), 119 (19.83%) in PCOS women and 34 (17.00%) in control (*p* = 0.378). Compared to the age, BMI and thyroid autoimmunity matched control group, TSH level of PCOS women is higher (2.29 ± 1.24 mU/L vs. 1.86 ± 0.90 mU/L, *p* < 0.001). Lipid profiles showed higher TG (1.19 (0.82–1.81) mmol/L vs. 1.01 (0.69–1.57) mmol/L, *p* = 0.002), TC (4.78 ± 0.99 mmol/L vs. 4.63 ± 0.91 mmol/L, *p* = 0.07), LDL-c (2.80 ± 0.80 mmol/L vs. 2.63 ± 0.76 mmol/L, *p* = 0.007) and apo B (0.95 ± 0.27 mmol/L vs. 0.88 ± 0.27 mmol/L, *p* = 0.007) were observed in PCOS population than controls. Furthermore, FSH (*p* = 0.012), ratio of LH/FSH, total T, free T, FAI, A_2_, and DHEAs were all significantly elevated in PCOS while SHBG was lower compared with controls (*p* < 0.001).

**Table 1:** Clinical and biochemical characteristics in PCOS patients and controls.

	**PCOS (*n* = 600)**					
**Characteristics^**a**^**	**nonHA (*n* = 229)**	**HA (*n* = 371)**	***P*^**c**^**	**Total**	**Control (*n* = 200)**	***P*^**d**^**
Age (years)	27.73 ± 4.90	27.63 ± 5.40	0.822	27.67 ± 5.21	28.26 ± 5.29	0.170
BMI (kg/m^2^)^b^	23.44 ± 4.70	28.26 ± 5.47	<0.001	26.44 ± 5.69	26.72 ± 5.37	0.537
Normal BMI	150	104	<0.001	252 (42.00)	77 (38.50)	0.516
Overweight	56	138		196 (32.67)	74 (37.00)	
Obese	23	129		152 (25.33)	49 (24.50)	
WC (cm)	79.99 ± 12.51	91.89 ± 14.16	<0.001	87.35 ± 14.73	86.52 (13.90)	0.188
WHR	0.85 ± 0.09	0.89 ± 0.07	<0.001	0.88 ± 0.08	0.87 ± 0.08	0.249
TSH (mIU/L)	2.16 ± 1.01	2.37 ± 1.37	0.045	2.29 ± 1.24	1.86 ± 0.90	<0.001
Phenotype			<0.001			
HA-O-PCO	0	190		190 (31.67)	NA	
O-HA	0	86		86 (14.33)	NA	
PCO-HA	0	95		96 (16)	NA	
PCO-O	229	0		228 (38)	NA	
HA phenotype	0	371	<0.001	371 (61.83)	NA	
AIT	53 (23.14)	66 (11.18)	0.11	119 (19.83)	34 (17.00)	0.378
TGAB positive	47 (20.32)	60 (16.11)	0.176	107 (17.83)	29 (14.50)	0.277
TPOAB positive	27 (11.13)	30 (8.09)	0.133	57 (9.50)	19 (9.50)	1.000
TG (mmol/L)	0.90 (0.67–1.37)	1.37 (1.01–2.02)	<0.001	1.19 (0.82–1.81)	1.01 (0.69–1.57)	0.002
Tch (mmol/L)	4.61 ± 0.96	4.88 ± 0.99	0.001	4.78 ± 0.99	4.63 ± 0.91	0.070
HDL (mmol/L)	1.51 ± 0.40	1.23 ± 0.32	<0.001	1.34 ± 0.38	1.43 ± 0.40	0.006
LDL (mmol/L)	2.57 ± 0.72	2.95 ± 0.81	<0.001	2.8 ± 0.80	2.63 ± 0.76	0.007
apoA (mg/L)	125.00 (41–252)	108.00 (48.95–259.00)	0.68	112.9 (46.23–253.45)	107.45 (41.00–259.50)	0.302
apoB (g/L)	0.85 ± 0.23	1.00 ± 0.28	<0.001	0.95 ± 0.27	0.88 ± 0.27	0.007
LH (IU/L)	8.72 ± 7.03	8.86 ± 6.01	0.787	8.81 ± 6.41	8.07 ± 7.31	0.201
FSH (IU/L)	6.40 ± 2.56	6.12 ± 3.50	0.205	6.23 ± 2.50	7.31 ± 5.48	0.012
LH/FSH	1.47 ± 1.15	1.50 ± 0.98	0.759	1.49 ± 1.05	1.21 ± 0.90	0.001
PRL (ug/L)	12.59 (8.96–19.36)	11.55 (8.76–16.02)	0.095	11.95 (8.96–16.92)	12.68 (9.40–16.62)	0.726
E_2_ (pmol/L)	183.00 (110.75–300.50)	168.00 (125.00–243.00)	0.338	174.00 (116.50–259.00)	172 (121.50–276.50)	0.956
*T* (nmol/L)	1.79 ± 0.75	2.53 ± 0.92	<0.001	2.25 ± 0.93	1.73 ± 0.75	<0.001
FT	25.35 ± 9.68	61.21 ± 21.89	<0.001	47.52 ± 25.21	27.05 ± 13.73	<0.001
FAI	3.73 (2.76–5.23)	12.46 (9.43–17.73)	<0.001	8.68 (4.66–14.17)	4.32 (2.74–5.68)	<0.001
mean ovary volume (ml^3^)	50.53 ± 24.12	19.62 ± 9.16	<0.001	10.41 ± 4.54	5.69 ± 1.26	<0.001
SHBG (nmol/L)	202.50 (149.25–258.75)	257.00 (183.75–321.25)	<0.001	24.2 (15.93–42.28)	39 (28.60–58.10)	<0.001
DHEAs (ng/mL)	3.78 ± 2.16	4.58 ± 2.26	<0.001	243.33 ± 104.59	198.31 ± 93.20	<0.001
A_2_ (ug/dL)	10.88 ± 4.92	10.10 ± 4.26	0.061	4.28 ± 2.26	2.94 ± 1.66	<0.001

*WC, waist circumference; WHR, waist to hip ratio; HA, hyperandrogenism; AIT, autoimmune-thyroiditis; FT, free testosterone; FAI, free androgen index; SHBG, sex hormone binding globulin; DHEAs, dehydroepiandrosterones*.

a *Continuous data are shown as mean ± sd or medians (IQR) and categorical variables as n (%)*.

b *Body mass index was calculated as weight (kg)/height (m)^2^*.

c *P-value for nonHA vs. HA PCOS*.

d *P-value for PCOS vs. control*.

### Clinical and Biochemical Characteristics of PCOS With or Without HA

The clinical and biochemical characteristics of PCOS with or without HA were summarized in Table 1. We found that HA subjects of PCOS have significantly higher BMI, WC, and WHR (all *p* < 0.001) compared to nonHA subjects. The prevalence of AIT is comparable in nonHA and HA subjects (23.14% vs. 11.18, *p* = 0.110). While TSH, TG, TC, LDL-c and apoB level increased from nonHA to HA group (all *p* < 0.05). As for the sex steroids, lower SHBG, higher total T, FAI, free T, DHEA-S, and A_2_ were observed, as expected.

### Clinical and Biochemical Characteristics of PCOS Women Grouped by TSH Level

As the controversy on treatment threshold mainly based on whether TSH value above 2.5 or 4.0 mIU/L, we further subdivided PCOS patient by TSH 2.5 mU/L (Table 2, Group 1, 0.25 ≤ TSH ≤ 2.5 mU/L; Group 2, 2.5 < TSH ≤ 5.0 mU/L) and TSH 4.0 mU/L (Table S1, Group S1, 0.25 ≤ TSH ≤ 4.0 mU/L; Group S2, 4.0 < TSH ≤ 5.0 mU/L). As shown in Table 2, when divided by TSH 2.5 mU/L we found women with relatively higher TSH level (Group 2) had significantly higher BMI, WC,TG, apoB, LDL-c, FT, FAI, and lower SHBG compared with Group 1 (all *p* < 0.05). Besides, higher prevalence of HA phenotype were observed in Group 2 (69.46% vs. 57.93%, *p* = 0.006). Furthermore, we analyzed those with AIT (Table S2), lipid profiles and HA prevalent were not different between Group 1 and Group 2 (all *p* > 0.05). We then subdivided PCOS patients by TSH level 4.0 mU/L, Group S2 had comparable HA phenotype, lipid profiles and sex steroids except lower SHBG compared to Group S1 (Table S1).

**Table 2:** Clinical and biochemical characteristics in PCOS patients grouped by TSH level.

**Characteristics^**a**^**	**Group 1^**b**^ (*n* = 397)**	**Group 2 (*n* = 203)**	***P***
Age (years)	27.76 ± 5.35	27.48 ± 4.94	0.533
BMI (kg/m^2^)^b^	26.06 ± 5.51	27.12 ± 5.98	0.032
Normal BMI	179 (45.09)	75 (36.95)	0.111
Overweight	126 (31.74)	68 (33.50)	
Obese	92 (23.17)	60 (29.56)	
WC (cm)	86.15 ± 14.67	89.69 ± 14.59	0.005
WHR	0.87 ± 0.08	0.88 ± 0.07	0.136
TSH (mU/L)	1.64 ± 0.50	3.57 ± 1.28	<0.001
**PHENOTYPE**			
HA-O-PCO	116 (21.17)	74 (40.22)	0.053
O-HA	56 (10.22)	30 (16.30)	
PCO-HA	59 (10.77)	37 (20.11)	
PCO-O	166 (30.29)	62 (33.70)	
HA phenotype	230 (57.93)	141 (69.46)	0.006
AIT	72 (18.14)	47 (23.15)	0.145
TGAB positive	65 (16.37)	42 (20.69)	0.191
TPOAB positive	31 (7.81)	26 (12.81)	0.048
TG (mmol/L)	1.15 (0.82–1.71)	1.34 (0.83–2.02)	0.048
Tch (mmol/L)	4.73 ± 0.97	4.88 ± 1.01	0.084
HDL (mmol/L)	1.33 ± 0.37	1.35 ± 0.39	0.569
LDL (mmol/L)	2.76 ± 0.79	2.9 ± 0.82	0.043
apoA	105.35 (44.00–244.25)	124 (50.75–266.25)	0.236
apoB	0.92 ± 0.26	0.98 ± 0.29	0.027
LH (IU/L)	8.94 ± 6.71	8.55 ± 5.79	0.462
FSH (IU/L)	6.38 ± 2.75	5.93 ± 1.89	0.021
LH/FSH	1.49 ± 1.06	1.49 ± 1.03	0.957
PRL (ug/L)	11.69 (8.96–16.99)	12.27 (8.96–16.78)	0.548
E_2_ (pmol/L)	173 (113.5–272.00)	178 (127.00–243.00)	0.985
*T* (nmol/L)	2.23 ± 0.93	2.29 ± 0.93	0.431
FT	45.7 ± 24.24	51.09 ± 26.71	0.016
FAI	8.18 (4.39–12.92)	10 (5.47–16.44)	0.001
A_2_ (ug/dL)	4.32 ± 2.26	4.20 ± 2.25	0.517
SHBG (nmol/L)	25.8 (16.95–43.75)	20.9 (13.90–35.60)	0.001
DHEAs (ng/mL)	237.64 ± 100.70	254.58 ± 111.27	0.061
mean ovary volume (ml^3^)	10.59 ± 4.78	10.10 ± 4.08	0.247

*WC, waist circumference; WHR, waist to hip ratio; HA, hyperandrogenism; AIT, autoimmune-thyroiditis; FT, free testosterone; FAI, free androgen index; SHBG, sex hormone binding globulin; DHEAs, dehydroepiandrosterones*.

a *Continuous data are shown as mean ± sd or medians (IQR) and categorical variables as n (%)*.

b *Group 1, 0.25 ≤ TSH ≤ 2.5 mU/L; Group 2, 2.5 < TSH ≤ 5.0 mU/L*.

### Relationships Between TSH Level, Lipid Profiles, and Androgen Related Parameters

**Figure 1:**
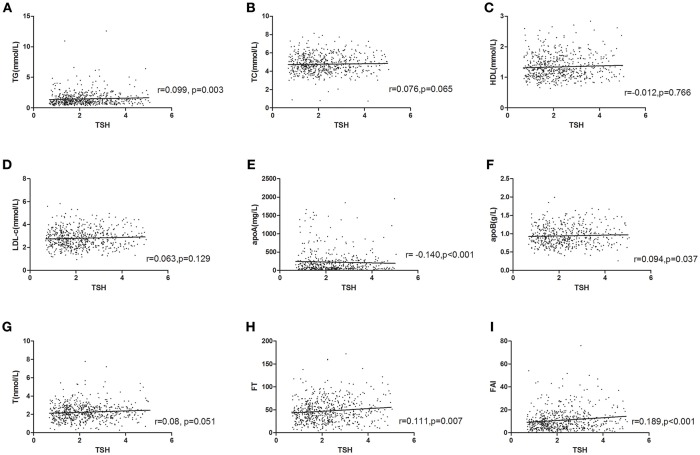
Relationship of TSH level with lipid and androgen parameters in PCOS. **(A)** TG (*r* = 0.099, *p* = 0.003); **(B)** TC (*r* = 0.076, *p* = 0.065); **(C)** HDL-c (*r* = −0.012, *P* = 0.766); **(D)** LDL-c (*r* = 0.063, *p* = 0.129); **(E)** apoA (*r* = −0.140, *p* < 0.001); **(F)** apoB (*r* = 0.094, *p* = 0.037); **(G)** total *T* (*r* = 0.08, *P* = 0.051); **(H)** free *T* (*r* = 0.111, *p* = 0.007); **(I)** FAI (*r* = 0.189, *P* < 0.001). *p*-value for Pearson (normal distribution)/Spearman's (skewed distribution) correlation tests.

We further analyze the relationships of TSH level with lipid and androgen related parameters in PCOS women. As shown in Figure 1, the level of TSH was positively associated with TG, (*r* = 0.099, *p* = 0.003),TC (*r* = 0.076, *p* = 0.065), LDL-c (*r* = 0.063, *p* = 0.129), apoB (*r* = 0.094, *p* = 0.037), total *T* (*r* = 0.08, *P* = 0.051), free *T* (*r* = 0.111, *p* = 0.007), FAI (*r* = 0.189, *P* < 0.001), and negatively associated with HDL-c (*r* = −0.012, *p* = 0.766), apoA (*r* = −0.140, *P* < 0.001).

### Analyses of Factors Associated With HA in Women With PCOS

Multinomial logistic regression analyses show that the risk for prevalent HA increased across TSH level after adjustment for age, BMI, WC, and LDL-C (Table 3). In multivariate-adjusted models, the ORs (95% CIs) for HA in the higher TSH level compared with the TSH below 2.5 mIU/L were 1.143 (95%CI: 1.033–2.016, *p* = 0.038). Furthermore, we conducted univariate binary logistic regression to evaluate the risk factors associated with HA in PCOS women (Table 4). We found HA was significantly associated with TSH, TG, TC, apo B, and LDL-c. In multivariate logistic regression analysis TSH > 2.5 mIU/L and TG remained significantly associated with the presence of HA (all *p* < 0.05).

**Table 3:** Associations of TSH level with HA in PCOS patients.

	**TSH level**			
	**Group 1**	**Group 2**	**95% CI**	***P* for trend**
HA	1	1.632	1.111–2.433	0.004
adjusted for age, BMI, WC	1	1.521	1.103–2.410	0.019
Multivariate-adjusted^*^	1	1.143	1.033–2.016	0.038

*Data are OR (95%CI) unless otherwise indicated*.

* *OR with corresponding 95% CI has been adjusted for age, BMI, WC, LDL-c*.

**Table 4:** Binary logistic regression with HA as output variable.

	**Univariate**				**Multivariate**			
**Variables**	**OR**	**95% CI**		***P***	**OR**	**95% CI**		***P***
TSH	1.815	1.216	2.887	0.004	1.573	1.024	2.416	0.038
TG	2.001	1.554	2.578	<0.001	1.343	1.01	1.785	0.043
TC	1.342	1.125	1.6	0.001	0.654	0.399	1.071	0.092
apoB	9.884	4.537	21.531	<0.001	2.327	0.511	10.601	0.275
LDL-c	1.485	1.148	1.923	0.003	1.595	0.832	3.056	0.159

*Data are OR (95%CI) unless otherwise indicated*.

## Discussion

In the current study, we show evidence to confirm PCOS women with relatively higher TSH level is associated with increased risk of HA phenotype from a large population. The elevated HA risk is evident after statistical correction for differences in age, BMI, thyroid autoimmunity, and increased across TSH level divided by 2.5 mU/L, suggesting the important role of TSH level and HA in PCOS women. To the best of our knowledge, this is the first study to investigate the association between TSH levels and HA risk in a large PCOS population with normal thyroid function in a single center.

In our cohort, serum TSH level is significant higher in PCOS population than age, BMI, and thyroid autoimmunity matched counterparties with normal thyroid function. TSH level is increased from nonHA to HA group (*p* = 0.04). Previous study revealed SCH and PCOS occur in conjunction had higher risk of metabolic disorders especially in lipid profiles. Total cholesterol (TC), triglyceride (TG) and LDL-c were higher in PCOS with SCH (11, 22, 23). Dittrich et al. (24) reported in a 103 PCOS cohort that women with TSH ≥ 2.5 mIU/L had a significantly higher BMI, fasting insulin, HOMA-IR, TC, FAI levels, and decreased SHBG level in comparison with those with TSH < 2.5 mIU/ L (24–26). This is accordance with our result, as in our euthyroid PCOS population, FT and FAI were significant higher in TSH ≥ 2.5 mIU/L group than their age-matched counterparts.

Among women of reproductive age, the prevalence of thyroid autoimmunity is 8–14% worldwide (3, 5). While in women from eastern China the prevalence is 21.4% (27), which is comparable with our cohort (20.13%). In addition, we find no difference between PCOS and controls (119/600, 19.83% vs. 34/200, 17.00%; *p* = 0.378). Women with positive thyroid antibodies have been reported to be at 2–3-fold higher risk of spontaneous miscarriage than those who test negative (28). The 2011 American Thyroid Association (ATA) and the 2012 Endocrine Society guidelines for the diagnosis and treatment of thyroid disease during pregnancy both declared that there was insufficient evidence to recommend for or against treating women with normal thyroid function experiencing sporadic or recurrent miscarriage or undergoing IVF-ET with levothyroxine (28, 29). Furthermore, ATA took a similar position in its 2017 guideline (18). Conflicting results were reported involving the relationship between TSH level and conception rates or time to pregnancy in infertility: Plowden et al. reported TSH ≥ 2.5 mIU/L has not been associated with increased time to pregnancy (30). Karmon et al. found preconceptional TSH ≥ 2.5 mIU/L is not associated with adverse intrauterine insemination outcomes in 1,477 euthyroid women (31). While in a large population including 11,254 Danish women, higher TSH level is associated with higher risk of not having children and not getting pregnant in age-adjusted and multi-adjusted models (32). In euthyroid women with unexplained infertility from Orouji et al. TSH ≥ 2.5 mIU/L has a higher risk of infertility (33). Whether levothyroxine treatment target for infertile women within 2.5mU/L is suitable for PCOS women was also still uncertain. Wang et al. (34) conducted a randomized clinical trial recently. Their result showed among women undergoing IVF-ET in China who had intact thyroid function and positive antithyroperoxidase antibodies, treatment with L-T4, compared with no levothyroxine treatment, did not reduce miscarriage rates or increase live-birth rates (34). So further clinical trials will be needed to investigate whether L-T4 treatment is suitable in PCOS population.

PCOS is the most common cause of female infertile in women during reproductive age. In our study, TSH value above 2.5 mU/L was associated with HA phenotype, which plays a key role in pathogenesis of PCOS. Gonadotropin-releasing hormone (GnRH) treatment has been shown to moderately increase TSH secretion in amphibians (35), suggesting that GnRH can modulate thyroid hormons at the pituitary level. As GnRH regulates the biosynthesis and secretion of both LH and FSH which were observed elevated in PCOS. Thyroid hormones modulate androgen biosynthesis through direct and indirect regulation of the expression and activity of steroidogenic enzymes associated synthesis (35). Recently, Flood et al. performed an *in silico* analysis of the promoter of several receptors and enzymes involved in both the androgen and hypothalamic-pituitary-thyroid axes (36). It was found that several putative androgen responsive elements (AREs) and thyroid responsive elements (TREs) were present in all of the androgen and thyroid hormone-related genes (36). This may give indication for there might be relatively subtle hypothyroidism in even normal PCOS population. If this were the case, supplementation of L-T4 before pregnancy in those PCOS women with HA phenotype may improve pregnancy outcomes. Further studies will be needed to confirm this hypothesis.

Several limitations of our study should be considered. These data come from cross-sectional analyses and thus have substantial limitations for drawing causal inferences. Prolonged follow-up was needed as a prospective study to evaluate the pregnant outcome in PCOS patients with different TSH level can provide better evidence on the relationship of TSH level and pregnancy.

## Conclusions

In conclusion, our results lend support to the postulation that the TSH level is associated with HA phenotype in PCOS women. We analyzed the TSH level in different PCOS phenotypes, we found TSH level is associated with HA phenotype in a well-characterized, large number of PCOS cohort. Our results show that TSH level, independently of age, BMI and thyroid autoimmunity, is associated with a higher HA prevalence in PCOS. Further studies are warranted to elucidate the role of TSH and HA phenotype in PCOS patients.

## Author Contributions

WL and TT designed the study. JC, YZ, YW, SL, JZ, LW, YJ, YD, HZ, YH, JM, and TT collected the data. JC, YZ, WL, and TT analyzed the data. JC and TT wrote the first draft of this report. All authors made critical revisions of the manuscript.

### Conflict of Interest Statement

The authors declare that the research was conducted in the absence of any commercial or financial relationships that could be construed as a potential conflict of interest.
